# Combination of glycopyrronium and indacaterol inhibits carbachol-induced ERK5 signal in fibrotic processes

**DOI:** 10.1186/s12931-017-0529-6

**Published:** 2017-03-11

**Authors:** Yukiko Namba, Shinsaku Togo, Miniwan Tulafu, Kotaro Kadoya, Kumi Yoneda Nagahama, Hikari Taka, Naoko Kaga, Akira Orimo, Xiangde Liu, Kazuhisa Takahashi

**Affiliations:** 1 Division of Respiratory Medicine, Juntendo University Faculty of Medicine & Graduate School of Medicine, 2-1-1 Hongo, Bunkyo-ku, Tokyo, 113-8421 Japan; 2 Research Institute for Diseases of Old Ages, Juntendo University Graduate School of Medicine, 2-1-1 Hongo, Bunkyo-ku, Tokyo, 113-8421 Japan; 3 Laboratory of Proteomics and Biomolecular Science, Research Support Center, Juntendo University Graduate School of Medicine, 2-1-1 Hongo, Bunkyo-ku, Tokyo, 113-8421 Japan; 4 Departments of Pathology and Oncology, Juntendo University School of Medicine, 2-1-1 Hongo, Bunkyo-ku, Tokyo, 113-8421 Japan; 5 Pulmonary Critical Care and Sleep Medicine, University of Nebraska Medical Center, Omaha, NE USA

**Keywords:** Acetylcholine, Extracellular-signal-regulated kinase 5 (ERK5), Long-acting β2-adrenergic receptor agonist, Long-acting muscarinic receptor antagonist, Transforming growth factor-β1

## Abstract

**Background:**

Airway fibrosis is one of the pathological features of chronic obstructive pulmonary disease (COPD), and recent studies revealed that acetylcholine plays an important role in the development of airway remodeling by stimulating proliferation and collagen synthesis of lung fibroblasts. This study was designed to examine the effects of a long-acting muscarinic receptor antagonist (LAMA) glycopyrronium and a long-acting β2 adrenergic receptor agonist (LABA) indacaterol on acetylcholine-mediated fibrotic responses in lung fibroblasts.

**Methods:**

After carbachol (CCh) or transforming growth factor-β1 (TGF-β1) exposure, the response to glycopyrronium and indacaterol was determined in vitro in fibroblasts isolated from mild-to-moderate COPD lung tissue. The ability of fibroblasts to mediate the contraction of collagen gels was assessed. The expression of α-smooth muscle actin (α-SMA) and the phosphorylation of extracellular-signal-regulated kinase 5 (ERK5) were determined by immunoblot. TGF-β1 was quantified by ELISA and acetylcholine was quantified by liquid chromatography tandem-mass spectrometry.

**Results:**

CCh stimulated fibroblast-mediated collagen gel contraction and α-SMA expression and TGF-β1 release by fibroblasts. Blockade of autocrine TGF-β1 attenuated CCh-mediated fibrotic responses, while TGF-β1 did not stimulate acetylcholine release. Glycopyrronium plus indacaterol significantly attenuated CCh- and TGF-β1-mediated fibrotic responses through inhibition of ERK5 phosphorylation. Notably, the magnitudes of CCh- and TGF-β1-stimulated gel contraction, CCh-induced TGF-β1 release, and ERK5 phosphorylation were greater in fibroblasts isolated from COPD subjects than in those from non-smokers.

**Conclusions:**

CCh induced TGF-β1 self-sustaining signaling loops by potentiating ERK5 signaling and promoted myofibroblast activity. This autocrine signaling mechanism may be an attractive therapeutic target to block the fibrotic response, which was modulated by the combination of glycopyrronium and indacaterol.

## Background

Persistent long-term airway inflammation contributes to airway remodeling and permanent alterations of the airway wall structure. Airway remodeling, including fibrosis, arises during the early stages of asthma and chronic obstructive pulmonary disease (COPD), and small airway fibrosis in COPD is associated with a greater decline in forced expiratory volume in 1 s (FEV1) than emphysema [1–3].

Resident lung fibroblasts are activated under chronic airway inflammation and differentiated into myofibroblasts, accompanied by an increased expression of α-smooth muscle actin (α-SMA) and augmented a persistence of contractile activity (3), contributing to irreversible airflow limitation [4]. Transforming growth factor (TGF)-β is a well-known fibrogenic mediator involved in COPD [5, 6], but recent evidence indicates the potential role of non-neuronal acetylcholine as a paracrine/autocrine mediator in the regulation of airway fibrosis [7]. A clinical study also showed that methacholine inhalations induced sub-epithelial collagen deposition and TGF-β immunoreactivity within the airway epithelium, which are suppressed by a short acting β2-agonist [8].

Acetylcholine is classically known as a neurotransmitter involved in airway muscle contraction and mucus secretion via the activation of muscarinic receptors, and the increased cholinergic tone of smooth muscle is a major therapeutic target of airflow limitation in COPD [9]. Notably, acetylcholine may not only be released from parasympathetic nerve, but also from airway epithelial cells and inflammatory cells [10, 11], and cholinergic stimuli increase the proliferation and collagen synthesis of human lung fibroblasts via extracellular signal-regulated kinase 1/2 (ERK1/2) [12–14] and enhance α-SMA expression and TGF-β1 production through ERK1/2 and Rho kinase [15]. Furthermore, fibroblasts express choline-acetyltransferase (ChAT), an enzyme regulating acetylcholine synthesis, and TGF-β1 stimulates ChAT expression [15, 16]. However, the acetylcholine release from fibroblasts has not yet been directly demonstrated. Additionally, extracellular-signal-regulated kinase 5 (ERK5), a member of the mitogen-activated protein kinase (MAPK) family, has recently garnered the attention as the positive regulator of TGF-β signaling, and inhibition of ERK5 ameliorated bleomycin-induced pulmonary fibrosis in mice [17], but the roles of ERK5 in airway fibrosis remain unknown.

Long-acting muscarinic antagonists (LAMAs) and long-acting β2 adrenergic receptor agonists (LABAs) are widely used as bronchodilators for maintenance therapy in COPD and asthma [18, 19]. QVA149, a fixed-dose combination of the LAMA, glycopyrronium (GLY), and the LABA, indacaterol (IND), is a novel, once-daily dual bronchodilator that demonstrated rapid and sustained bronchodilation in patients with moderate-to-severe COPD [20]. However, whether the combination of LAMA and LABA modifies airway fibrosis remains unclear, particularly during early/mild COPD.

In this study, we investigated the effect of GLY and IND in regulating carbachol (CCh) and TGF-β1-induced ERK5 activation during the fibrotic processes in lung fibroblasts isolated from patients with COPD. In addition, we explored a possible link between CCh and TGF-β1 signaling.

## Methods

### Materials

Dulbecco’s Modified Eagle’s Medium (DMEM) was purchased from Wako (Osaka, Japan) and FCS from Sigma-Aldrich (St. Louis, MO, USA). Novartis Pharma AG (Basel, Switzerland) provided GLY and IND. CCh was from Sigma-Aldrich and TGF-β1 from R&D Systems (Minneapolis, MN, USA). ERK5 inhibitor, BIX02189, and activin receptor–like kinase 5 (ALK5) inhibitor, SB431542, were from Selleckchem (Houston, TX, USA) and ERK1/2 inhibitor, PD98059, from Calbiochem (La Jolla, CA, USA). Rho-associated coiled-coil forming kinase/Rho binding kinase inhibitor, Y-27632, was from Wako. The primary antibodies were anti-ERK5 (Cell Signaling Technology, Danvers, MA, USA: Catalogue no. #3372), anti–α-smooth muscle actin (α-SMA) (Sigma-Aldrich: Catalogue no. A2547), and anti- ChAT antibody (Merck KGaA, Darmstadt, Germany: Catalogue no. AB144P).

### Isolation and primary culture of lung fibroblasts

Human primary lung parenchymal fibroblasts from patients undergoing lung resection for localized tumor were cultured as described previously [21]. Briefly, the portion of lung parenchymal tissue that was as distal from any tumors as possible and free of the pleural surface was minced and cultured in DMEM supplemented with 10% FCS, 100 μg/ml penicillin, 250 μg/ml streptomycin, and 1 μg/ml amphotericin B in a humidified atmosphere of 5% CO_2_. Cells were assessed in passage 2 after primary culture and displayed the typical fibroblast morphology and were positive for vimentin and negative for cytokeratin staining. Lung fibroblasts from 22 patients: 11 non-smokers without clinical airway symptom or lung functional abnormalities, 11 smokers with mild-to-moderate COPD according to the Global Initiative for Chronic Obstructive Lung Disease classification (GOLD 1 and 2) were obtained. Patients with a history of asthma and marked eosinophil infiltration in airways of resected lungs were excluded. The Institutional Review Board at the Juntendo University School of Medicine approved the procedures. All patients provided written, informed consent (No. 2013051).

### Cell culture

Human fetal lung fibroblasts (HFL1; catalogue number CCL-153) and A549 (catalogue number CCL-185) were purchased from the American Type Culture Collection (Manassas, VA, USA). Lung fibroblasts and A549 cells were cultured in DMEM supplemented with 10% FCS, 100 μg/ml penicillin, 250 μg/ml streptomycin, and 1 μg/ml amphotericin B in a humidified atmosphere of 5% CO2. Sub-confluent cells were removed from the dishes by 0.05% trypsin-EDTA (Wako, Osaka, Japan). For three-dimensional collagen gel contraction and ELISA, primary lung fibroblasts were used at the fourth to sixth passages after isolation to exclude the effect of differences in passage number and culturing conditions.

### Type I collagen preparation

Type I collagen (rat tail tendon collagen, RTTC) was extracted from rat tail tendons as previously described [22].

### Three-dimensional collagen gel contraction assay

The effect of CCh and TGF-β1 on fibroblast-mediated collagen gel contraction was assessed in the presence/absence of GLY and IND, BIX02189, and SB431542, using a modification of a previously described method [23]. Briefly, sub-confluent fibroblasts were deprived of serum for 24 h, detached with 0.05% trypsin-EDTA, and resuspended in serum free DMEM (SF-DMEM). The cell number was then counted with a Coulter Counter (Beckman Coulter, Inc., Fullerton, CA, USA). Collagen gels were prepared by mixing the appropriate amount of RTTC, distilled water, 4× concentrated DMEM, and cell suspension. The final mixture was 0.75 mg/ml of collagen, 3 × 10^5^ fibroblasts/ml gel, and a physiologic ionic strength of 1× DMEM, and a pH of 7.4. A portion of the gel solution (500 μl) was then casted into each well of a 24-well tissue culture plate with a 1.9 cm^2^ growth area (Corning, Costar, NY, USA). Gelation occurred in 20 min at room temperature, after which the gels were released from the surface of the culture well using a sterile spatula. They were then transferred into 60 mm tissue culture dishes (three gels in each dish) containing 5 ml of SF-DMEM with or without designated reagents and incubated at 37 °C, 5% CO2 for 3 days. The ability of fibroblasts to contract the floating gels was determined by quantifying the area of the gels daily using an LAS4000 image analyzer (GE Healthcare Bio-Science AB, Uppsala, Sweden). Data are expressed as the percentage of gel area compared with the original gel size. Contraction is then expressed as a decrease in gel area.

### Measurement of TGF-β1 release

TGF-β1 release in the monolayer culture media was measured by ELISA. Sub-confluent lung fibroblasts on a 6-well plate were deprived of serum for 24 h and stimulated with CCh in the presence or absence of GLY or/and IND. Supernatants were harvested after 24 h, frozen, and stored at − 80 °C until assay. TGF-β1 production from lung fibroblasts was determined by human TGF-β1 Immunoassay (R&D Systems; Minneapolis, MN, USA) according to the manufacturer’s instructions.

### Determination of acetylcholine by LC–MS/MS

Acetylcholine (ACh) in the culture supernatants was analyzed by liquid chromatography tandem-mass spectrometry (LC-MS/MS). Sub-confluent lung fibroblasts on a 6-well plate were deprived of serum for 24 h and stimulated with TGF-β1. After 24 h, supernatants were transferred to 1.5 ml tubes in the presence of 1 μM rivastigmine (Sigma-Aldrich) to prevent ACh degradation. For ACh measurement, 5 μl of the aliquots were injected directly into the LC-MS/MS system. LC-MS/MS analysis were performed on Gilson HPLC system (Gilson, Villiers-le-Bel, France) connected to TSQ Quantum Ultra AM mass spectrometer (Thermo Fisher Scientific). ACh was analyzed in the positive mode and selected reaction monitoring (SRM) analysis was carried out. MS conditions were as follows: The collision energy was 15 eV and the Argon gas as collision gas was set at 1mTorr. The spray voltage was 3.5 kV; the capillary temperature was set at 300 °C. The nitrogen flow rate was 25 units for sheath gas and 5 units for auxiliary gas. Discovery HS F5-3 2.1 mm i.d × 150 mm, 3 μm column (Supelco, Bellefonte, PA, USA) was used for LC separation. The column temperature was set at 40 °C. The mobile phase A consisted of 0.1% formic acid in water and the mobile phase B consisted of 0.1% formic acid in 80% acetonitrile (v/v). The elution program started at 0% B and increased to 40% for 20 min. The flow rate was set to 0.2 ml/min. To obtain the internal standard report, acetylcholine (Wako, Osaka, Japan) was added to the SF-DMEM as control.

### Immunoblotting

The expression of α-SMA and ChAT as well as ERK5 phosphorylation were evaluated by immunoblotting analysis of cell lysates by using anti-α-SMA antibody (1: 5000), anti-ChAT antibody (1: 1000), and anti-ERK5 antibody (1: 1000). ERK5 phosphorylation can be detected by the presence of a band with a slower electrophoretic mobility shift [24]. Lung fibroblasts were seeded on 60 mm dish at a density of 5 × 10^4^/ml and cultured for 24 h. The medium was then changed to SF-DMEM for 24 h, following which cells were stimulated with CCh and TGF-β1 in the presence or absence of inhibitors for 48 h. After treatment, the cells were washed with ice-cold PBS and lysed with radioimmunoprecipitation assay lysis buffer (Wako, Osaka, Japan) supplemented with protease inhibitor (Thermo Fisher Scientific) and phosphatase inhibitor cocktail (Thermo Fisher Scientific). The lysates were briefly sonicated and then centrifuged at 12,000 rpm for 10 min. The protein concentration in the supernatants was measured by BCA Protein Assay Kit (Thermo Fisher Scientific). Lysates were solubilized in 4× Laemmli Sample Buffer (Bio-RAD). Equal amounts of protein were loaded into each lane, separated in 7.5–10% SDS-PAGE and transferred to polyvinylidene difluoride membrane. Membranes were blocked for 1 h or overnight in blocking reagent. The membrane was probed with antibodies to ERK5, ChAT, α-SMA, and β-actin. Bound antibodies were visualized using peroxidase-conjugated second antibodies and enhanced chemiluminescence with LAS4000 image analyzer (GE, Healthcare Bio-Science AB) and luminescent images were analyzed with an ImageQuant TL (GE Healthcare Bio-Science AB).

### Statistical analysis

Results are expressed as the means ± SEM. Grouped data were evaluated by one-way analysis of variance (ANOVA), corrected by the Bonferroni’s test. Samples that appeared different within a series were further assessed by Student’s *t*-test. Comparisons between non-smokers and patients with COPD were performed using the unpaired, two-tailed Mann–Whitney test. For evaluation of experimental studies within a group where paired samples were available, the Wilcoxon test was used. For these comparisons, each subject was considered as an individual point. *P* values less than 0.05 were considered significant. All data were analyzed using GraphPad Prism 6 (GraphPad Software, San Diego, CA, USA).

## Results

### Clinical and demographic features

The clinical and demographic features of the patients are shown in Table 1. The two groups were similar in age and sex. Patients with COPD were classified according to the GOLD consensus report criteria as GOLD 1 (*n* = 3) or GOLD 2 (*n* = 8). The lung function differed significantly between the two groups. As expected, patients with COPD had lower FEV_1_/FVC and FEV_1_% predicted value.

**Table 1:** Clinical and demographic features of patients

	No. of Non-smokers Patients	Mean ± SD	No. of Patients with COPD		Mean ± SD
Age, y	11	64 ± 8		11	66 ± 9
Sex, no. male/female	5/6			8/3	
Smoking status					
Never smoked	11			0	
Former/Current	0/0			4/7	
Pack-years	11	0		11	52.2 ± 32
GOLD 0/1/2/3/4	11/0/0/0/0			0/3/8/0/0	
FEV1/FVC, %	11	77.2 ± 5		11	60.4 ± 9*
FEV1, % predicted value	11	107.2 ± 10		11	73.2 ± 15*
VC, L	11	3.5 ± 1		11	3.6 ± 1

*COPD* chronic obstructive pulmonary disease, *GOLD* global initiative for chronic obstructive lung disease

*p < 0.001 compared with control

### Effect of GLY and/or IND on CCh or TGF-β1-induced collagen gel contraction and α-SMA expression

**Fig. 1:**
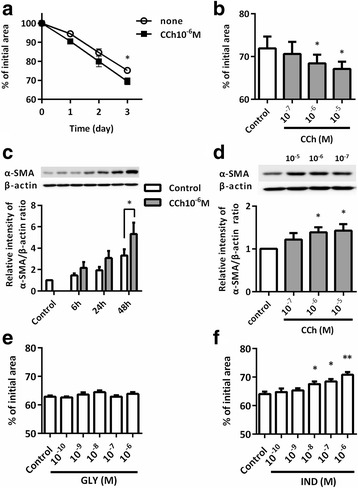
Effect of CCh, GLY or IND on collagen gel contraction and α-SMA expression in HFL-1 cells. Cells were grown to sub-confluence and deprived of serum for 24 h. Fibroblast-populated collagen gels were released into serum-free DMEM and measured the gel size daily by an image analyzer. **a** Gel size was measured in the presence of varying concentrations of CCh (**b**), GLY (**e**) or IND (**f**) on day3. Vertical axis, gel size expressed as % of initial size; Horizontal axis, conditions. All values represent the mean ± SEM in at least 3 separate experiments. HFL-1 cells were grown to sub-confluence in monolayer culture and stimulated with CCh at the indicated times or for 48 h at the indicated concentrations. After incubation, total protein was extracted and western blot analysis was performed with specific antibodies for α-SMA. Vertical axis: relative intensity of α-SMA/β-actin ratio. Horizontal axis: time (**c**) and concentration of CCh (**d**). All values represent the mean ± SEM in at least 3 separate experiments. *P < 0.05, **P < 0.01 compared with control

To investigate the effect of acetylcholine on tissue fibrotic response, fibroblast-mediated collagen gel contraction assay was used as an in vitro model of tissue remodeling. α-SMA expression was determined as one of contractile stress fibers, leading to gel contraction. Acetylcholine is easily metabolized by acetylcholinesterase. Therefore, we selected CCh as the long acting cholinergic agonist to stimulate muscarinic receptors. The expression of muscarinic receptor (M1-3) and β2 adrenergic receptor in HFL1 cells were confirmed using western blot analysis (data not shown). CCh significantly augmented HFL1-mediated collagen gel contraction and α-SMA expression in a time and concentration-dependent manner (*P* < 0.05 at 10^−6^ M, Fig. 1a-d). CCh were widely used as the concentration of 10^−5^M in laboratory setting, but previous study reported human normal bronchi contained 2–3 nmol/g of acetylcholine [25], suggesting the possibility of lower concentration than 10^−5^M acetylcholine. Therefore, we decided to select 10^−6^ M CCh in our experiments.

**Fig. 2:**
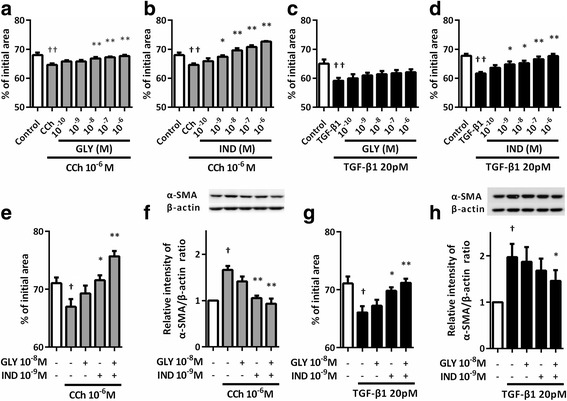
Effect of GLY and/or IND on CCh- or TGF-β1-augmented gel contraction and α-SMA expression in HFL1 cells. Sub-confluent fibroblasts were placed in serum-free (SF)-DMEM for 24 h. Cells were pre-treated with GLY and IND for 30 min, casted into collagen gels, and released into SF-DMEM containing various concentrations of GLY and/or IND with or without CCh (**a**, **b**, **e**) or TGF-β1 (**c**, **d**, **g**). Gel size was measured on day 3. Vertical axis: gel size expressed as a percentage of control. Horizontal axis: conditions. All values represent the mean ± SEM of three separate experiments, each performed in triplicate. For α-SMA assay, cells were grown to sub-confluence in monolayer culture and serum deprived for 24 h. Cells were then stimulated with CCh or TGF-β1 in the presence or absence of GLY and/or IND. After 48 h of incubation with CCh and 24 h of incubation with TGF-β1, cells were harvested for immunoblotting using a α-SMA antibody (**f**, **h**). Vertical axis, relative intensity of α-SMA/β-actin ratio; Horizontal axis, conditions. Representative immunoblots are shown at the top of each panel. All values represent the mean ± SEM in three different strains. ^†^ *P* < 0.05, ^† †^ *P* < 0.01, compared with solvent control. *P < 0.05, **P < 0.01, compared with stimulus

We examined the effect of GLY or IND on HFL1-mediated collagen gel contraction. IND inhibited gel contraction in a dose-dependent manner (*P* < 0.05 at concentrations of 10^−8^ to 10^−6^ M IND compared to control, Fig. 1f). However, GLY had no inhibitory effect (Fig. 1e). GLY or IND inhibited CCh-augmented gel contraction in a dose-dependent manner (*P* < 0.05 at concentrations of 10^−8^ to 10^−6^ M GLY and of 10^−9^ to 10^−6^ M IND compared with control, Fig. 2a and b). IND inhibited gel contraction in the presence of TGF-β1, which is known as a key mediator in the development of fibrosis and strongly stimulates collagen gel contraction and α-SMA expression in fibroblasts [26, 27] (*P* < 0.05 at concentrations of 10^−9^ to 10^−6^ M IND compared to TGF-β1 alone, Fig. 2d). Nevertheless, GLY had no effect on TGF-β1-mediated gel contraction (Fig 2c). In clinical settings, the maximum concentration in plasma of GLY 50 μg and IND 150 μg which were administered by inhalation once daily for 14 days were 216 pg/ml (0.5 nM) and 438.6 pg/ml (1.1 nM), respectively [28, 29]. However, there are no direct evidence for lung concentration after inhalation, we decided to select 10^−8^ M GLY and 10^−9^ M IND to access the pharmacological interaction between GLY and IND. Next, we investigated the effect of a combination of GLY and IND on CCh- or TGF-β1-mediated gel contraction in HFL1 cells. The combination of GLY 10^−8^ M and IND 10^−9^ M significantly suppressed CCh-augmented collagen gel contraction (CCh; 67.0 ± 1.3% vs. CCh + GLY + IND; 76.0 ± 1.0%: *P* < 0.01) as well as TGF-β1-augmented gel contraction (TGF-β1; 66.1 ± 1.1% vs. TGF-β1 + GLY + IND; 71.2 ± 0.7%: *P* < 0.01) (Fig. 2e and g). The combination of 10^−8^ M GLY and 10^−9^ M IND significantly suppressed CCh- or TGF-β1-induced α-SMA expression (*P* < 0.01 and *P* < 0.05, respectively) (Fig. 2f and h).

### Phenotypic differences in fibroblasts from non-smokers and patients with COPD on CCh- or TGF -β1-induced collagen gel contraction

**Fig. 3:**
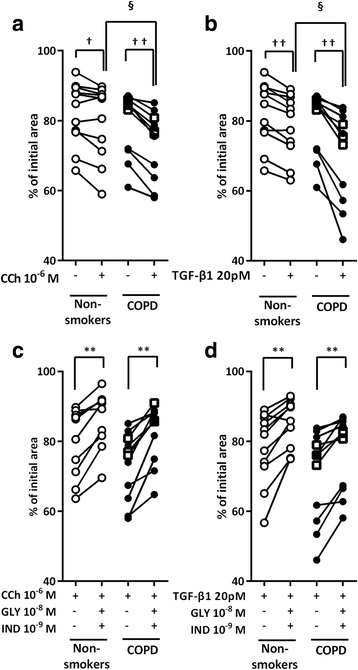
CCh- or TGF-β1-augmented gel contraction was attenuated by GLY and IND in primary lung fibroblasts. After 24 h of SF-DMEM culture, lung fibroblasts from non-smokers or patients with COPD were pre-treated with GLY or IND for 30 min, casted into collagen gels and released into SF-DMEM in the presence or absence of CCh (**a**), TGF-β1 (**b**), with or without GLY/IND (**c**, **d**). Gel size was measured on day 3. Vertical axis: gel size expressed as a percentage of control. Horizontal axis: conditions. Each pair of symbols connected by a line represents data of an individual cell strain isolated from non-smokers or patients with COPD. Cells from non-smokers are indicated by open circles, cells from patients with COPD (GOLD I) by squares, and cells from patients with COPD (GOLD II) by closed circles. ^†^ *P* < 0.05, ^† †^ *P* < 0.01, compared with control. *P < 0.05, **P < 0.01, compared with stimulus. ^§^ *P* < 0.05, compared with non-smokers and COPD lung fibroblasts in the same stimulus group

We next examined the phenotypic features of fibroblasts from non-smokers and patients with COPD in response to CCh and TGF-β1 stimulation. CCh significantly augmented collagen gel contraction mediated by the fibroblasts isolated from non-smokers (control; 82.0 ± 2.7% vs. CCh; 80.0 ± 3.2%; *P* < 0.05) or patients with COPD (control; 79.0 ± 2.8% vs. CCh; 73.0 ± 2.9%; *P* < 0.01) (Fig. 3a). Similarly, TGF-β1 also augmented collagen gel contraction mediated by the fibroblasts isolated from non-smokers (control; 82.0 ± 2.7% vs. TGF-β1; 78.8 ± 2.7%; *P* < 0.01) or patients with COPD (control; 79.0 ± 2.8% vs. TGF-β1; 70.1 ± 4.0%, *P* < 0.01) (Fig. 3b). It is noteworthy that the magnitudes of collagen gel contraction in response to CCh or TGF-β1 stimulation were greater in the fibroblasts isolated from patients with COPD than in those from non-smokers. Combination of GLY 10^−8^ M and IND 10^−9^ M significantly suppressed CCh- or TGF-β1-augmented collagen gel contraction in the fibroblasts isolated from both non-smokers and COPD subjects (*P* < 0.01) (Fig. 3c and d).

### Effect of CCh on the release of endogenous TGF-β1

**Fig. 4:**
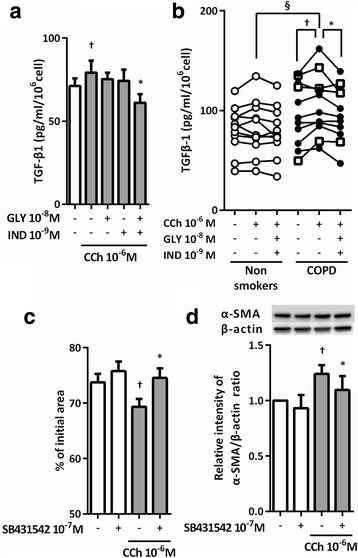
Effect of CCh-mediated endogenous TGF-β1 release in lung fibroblasts. Sub-confluent HFL1 cells (**a**) and primary lung fibroblasts (**b**) were cultured in SF-DMEM for 24 h in the presence or absence of CCh with or without GLY and/or IND. After 24 h of incubation, supernatants were harvested and used for TGF-β1 quantification. Vertical axis: TGF-β1 production expressed as amount per cells. Horizontal axis: conditions. Each pair of symbols connected by a line represents data of an individual cell strain isolated from non-smokers or patients with COPD. Cells from non-smokers are indicated by open circles, cells from patients with COPD (GOLD I) by squares, and cells from patients with COPD (GOLD II) by closed circles. **c** Sub-confluent HFL1 cells were cultured in SF-DMEM for 24 h. Cells were casted into collagen gels in the presence of CCh with or without SB431542, a selective inhibitor of the TGF-β1 receptor, ALK5. Gel size was measured on day 3. Vertical axis: gel size expressed as a percentage of control. Horizontal axis: conditions. All values represent the mean ± SEM of three separate experiments, each performed in triplicate. **d** HFL1 cells were grown to sub-confluence in monolayer culture and serum deprived for 24 h. Cells were stimulated with CCh in the presence or absence of SB431542. After 48 h of incubation, cells were harvested for the detection of α-SMA by immunoblotting. Vertical axis, relative intensity of α-SMA/β-actin ratio; Horizontal axis, conditions. All values represent the mean ± SEM of the three different strains. ^†^ *P* < 0.05, compared with solvent control. *P < 0.05, compared with stimulus. ^§^ *P* < 0.05, compared with non-smokers and COPD lung fibroblasts in the same stimulus group

We assessed the effects of CCh on the release of endogenous TGF-β1 and the effect of GLY and IND on the release of endogenous TGF-β1 by fibroblasts. CCh stimulated TGF-β1 release by HFL1 cells. The effect was significantly suppressed by a combination of GLY 10^−8^ M and IND 10^−9^ M (Fig. 4a). CCh-stimulated TGF-β1 release was significantly higher in the primary lung fibroblasts from patients with COPD than in those from non-smokers (*P* < 0.05). The suppression of CCh-stimulated TGF-β1 release by GLY 10^−8^ M plus IND 10^−9^ M was significantly stronger in the fibroblasts isolated from patients with COPD than in those from non-smokers (*P* < 0.05, Fig. 4b).

This result suggested that CCh-induced collagen gel contraction and α-SMA expression might be mediated through a TGF-β1 autocrine release/paracrine mechanism. Moreover, CCh-induced collagen gel contraction and α-SMA expression were partially suppressed by SB431542, a selective inhibitor of the TGF-β1 receptor, ALK5 (Fig. 4c and d).

### Quantification of acetylcholine release from lung fibroblasts

**Fig. 5:**
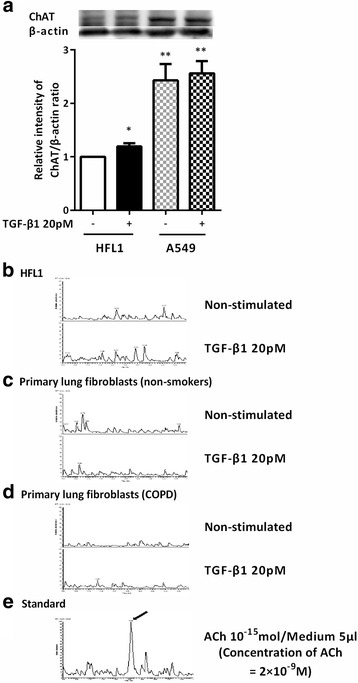
Determination of acetylcholine release by TGF-β1-stimulated lung fibroblasts by using LC-MS/MS. HFL1 and A549 cells were grown to sub-confluence in monolayer culture and serum deprived for 24 h. Cells were stimulated with TGF-β1 and harvested for the detection of ChAT by immunoblotting. Vertical axis, relative intensity of ChAT/β-actin ratio; Horizontal axis, conditions. Representative immunoblots are shown at the top of the panel. All values represent the mean ± SEM of the three different strains (**a**). HFL1 cells (**b**) and primary lung fibroblast from non-smokers (**c**) and patients with COPD (**d**) were grown to sub-confluence and cultured in SF-DMEM with or without TGF-β1 for 24 h. Supernatants were then harvested into a 1.5 ml tube and acetyl- and butyrylcholinesterase inhibitor (1 μM rivastigmine) was added to prevent acetylcholine degradation before storing at − 80 °C until assay. For the acetylcholine assay, the aliquots were thawed immediately before analysis and 5 μl of the samples was injected directly into the LC-MS/MS system. Data shown are representative chromatograms (m/z 86.910–87.910), performed in each group (*n* = 3). To obtain a standard report, acetylcholine was added to SF-DMEM. The arrow indicates the peak of acetylcholine (**e**). Vertical axis, signal intensity; Horizontal axis, retention time. *P < 0.05, **P < 0.01, compared with control

Acetylcholine is mainly synthesized from choline and acetyl-CoA by ChAT. Therefore, ChAT protein level in HFL1 and A549 cells was semi-quantitatively assessed by immunoblot. A549 cells were shown to release acetylcholine [30]. ChAT was detected in both HFL1 and A549 cells. It was slightly, but significantly, stimulated by TGF-β1 in HFL1 cells (Fig. 5a, *P* < 0.05). Interestingly, ChAT protein was significantly higher in A549 cells under basal conditions compared to that in HFL1 cells and TGF-β1 did not alter ChAT protein level in A549 cells (Fig. 5a).

Next, we measured acetylcholine in the supernatants of cultured fibroblasts by LC-MS/MS. Acetylcholine was not detectable in the culture medium of HFL1 cells or primary lung fibroblasts from non-smokers and patients with COPD (Fig. 5b-d), although 2 × 10^−9^ M acetylcholine could be detected as the positive control (Fig. 5e). Furthermore, TGF-β1 did not stimulate acetylcholine release in fibroblasts (Fig. 5b-d). This result indicated that the release of acetylcholine from fibroblasts might be only a modest level.

### Effect of GLY and IND on CCh or TGF-β1 induced ERK5 phosphorylation

**Fig. 6:**
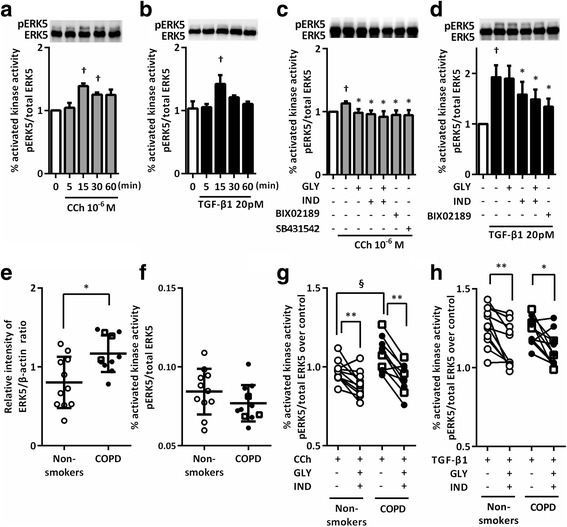
GLY and IND suppressed ERK5 phosphorylation in response to CCh or TGF-β1 in lung fibroblasts. Sub-confluent HFL1 cells were cultured in SF-DMEM with or without CCh (**a**) or TGF-β1 (**b**) for the indicated times. ERK5 phosphorylation was determined by immunoblotting. The bands with slower electrophoretic mobility corresponded to the activated kinase. Sub-confluent HFL1 cells (**c**, **d**) or primary fibroblasts (**e**-**h**) were serum deprived for 24 h followed by stimulation with or without TGF-β1 or CCh in the presence or absence of GLY and/or IND, SB431542 and ERK5 inhibitor (BIX02189) for 48 h. Cells were harvested and immunoblotted for ERK5. Each pair of symbols connected by a line represents data from an individual cell strain. Cells from non-smokers are indicated by open circles, cells from patients with COPD (GOLD I) by squares, and cells from patients with COPD (GOLD II) by closed circles. Vertical axis, the fold increase in activated kinase (p-ERK5) against total ERK5 over control or relative intensity of ERK5/β-actin ratio; Horizontal axis, times or conditions. All values represent the mean ± SEM. ^†^ *P* < 0.05, compared with solvent control. *P < 0.05, **P < 0.01, compared with stimulus. ^§^ *P* < 0.05, compared with non-smokers and COPD lung fibroblasts in the same stimulus group

We explored the role of ERK5 in mediating CCh and TGF-β1 signaling and the effect of GLY and IND on ERK5 phosphorylation. CCh and TGF-β1 induced ERK5 phosphorylation (*P* < 0.05, Fig. 6a-d). GLY plus IND and BIX02189, a specific MEK5-ERK5 inhibitor, significantly suppressed CCh- or TGF-β1-induced ERK5 phosphorylation (*P* < 0.05, Fig. 6c and d). Moreover, CCh-induced ERK5 phosphorylation was suppressed by SB431542 (Fig. 6c), suggesting that TGF-β1 autocrine release was involved in CCh signaling. Notably, the basal level of ERK5 expression was significantly higher in fibroblasts from patients with COPD than in those from non-smokers (*P* < 0.05, Fig. 6e), although ERK5 phosphorylation did not differ between the two groups (Fig. 6f). However, ERK5 phosphorylation in response to CCh stimulation was higher in fibroblasts from patients with COPD than in those from non-smokers (*P* < 0.05, Fig. 6g). In contrast, in response to TGF-β1 stimulation, ERK5 phosphorylation was similar in fibroblasts from patients with COPD and non-smokers (Fig. 6h). GLY plus IND significantly suppressed CCh- or TGF-β1-induced ERK5 phosphorylation in fibroblasts regardless of their origin (*P* < 0.05, Fig. 6g and h).

### Blockade of ERK5 phosphorylation resulted in the suppression of collagen gel contraction and α-SMA expression

**Fig. 7:**
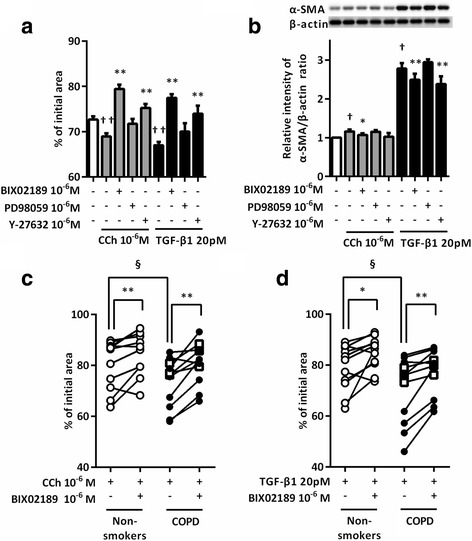
Inhibition of ERK5 resulted in the suppression of CCh- or TGF-β1-stimulated gel contraction and α-SMA expression in lung fibroblasts. Sub-confluent HFL1 cells were pre-treated with ERK5 inhibitor (BIX02189), ERK1/2 inhibitor (PD98059), and Rho kinase inhibitor (Y27632) for 30 min followed by being casted into collagen gels and treated with CCh or TGF-β1. Gel size was measured on day 3. Vertical axis: gel size expressed as a percentage of control. Horizontal axis: conditions. All values represent the mean ± SEM in three separate experiments, each performed in triplicate (**a**). Sub-confluent HFL1 cells were serum deprived for 24 h followed by stimulation with CCh or TGF-β1 for 48 h. Cells were then harvested for α-SMA immunoblotting. Vertical axis, relative intensity of α-SMA/β-actin ratio; Horizontal axis, conditions. All values represent the mean ± SEM in the three different strains (**b**). Lung fibroblasts from non-smokers and subjects with COPD were grown to sub-confluence and pre-treated with BIX02189 for 30 min followed by being casted into collagen gels and released into SF-DMEM with CCh (**c**) or TGF-β1 (**d**) in the presence or absence of BIX02189. Gel size was measured on day 3. Vertical axis: gel size expressed as a percentage of control. Horizontal axis: conditions. Each pair of symbols connected by a line represented data from an individual cell strain and the mean of three separate experiments. Cells from non-smokers are indicated by open circles, cells from patients with COPD (GOLD I) by squares, and cells from patients with COPD (GOLD II) by closed circles. ^†^ *P* < 0.05, ^††^ *P* < 0.01, compared with solvent control. *P < 0.05, **P < 0.01, compared with stimulus. ^§^ *P* < 0.05, compared with non-smokers and COPD lung fibroblasts in the same stimulus group

We assessed whether ERK5 blockade altered CCh- or TGF-β1-augmented collagen gel contraction and α-SMA expression in fibroblasts. Pretreatment with BIX02189 or Y-27632, a Rho kinase inhibitor, resulted in the significant reduction of CCh- or TGF-β1-augmented collagen gel contraction and α-SMA expression in HFL1 cells. However, PD98059, an ERK1/2 inhibitor did not significantly attenuate CCh- or TGF-β1-augmented collagen gel contraction and α-SMA expression (Fig. 7a and b). CCh-augmented collagen gel contraction by fibroblasts from patients with COPD or non-smokers was significantly inhibited by BIX02189 (*P* < 0.01, Fig. 7c). However, BIX02189 suppression of TGF-β1-augmented collagen gel contraction was more significant in fibroblasts from patients with COPD than in those from non-smokers (*P* < 0.01 and *P* < 0.05, respectively) (Fig. 7d).

**Fig. 8:**
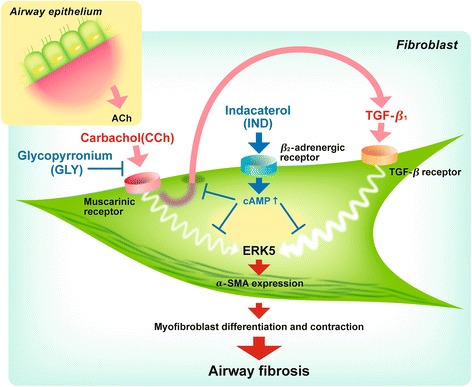
Schematic illustration of CCh-induced endogenous TGF-β1 signaling loop in COPD lung fibroblasts. Carbachol (CCh) induces ERK5 activation directly or indirectly through stimulating endogenous transforming growth factor-β1 (TGF-β1) release. Glycopyrronium (GLY) directly blocks CCh binding to its receptor, while indacaterol (IND) interrupts CCh signaling pathway or TGF-β1-induced ERK5 activation. Through these mechanisms, GLY plus IND blocked CCh- or TGF-β1-augmented collagen gel contraction and α-SMA expression

## Discussion

In the current study, we demonstrated for the first time that GLY and IND additively attenuated lung fibroblast-mediated contraction of collagen gels in response to CCh or TGF-β1. Interestingly, compared to lung fibroblasts isolated from non-smokers, the response of lung fibroblasts from patients with mild to moderate COPD to CCh- or TGF-β1-induced collagen gel contraction as well as to CCh-stimulated TGF-β1 release was higher. CCh or TGF-β1 induced ERK5 phosphorylation and inhibition of ERK5 phosphorylation by GLY plus IND resulted in the reduction of CCh- or TGF-β1-augmented collagen gel contraction and α-SMA expression in lung fibroblasts (Fig. 8). This functional involvement of a non-neuronal cholinergic system provide new insights into the pathogenesis of COPD and treatment strategy combining inhaled IND and GLY to patients with developing airway fibrosis in early stage of COPD.

Accumulating evidence indicates that acetylcholine in the airway may not only be released from parasympathetic nerve, but also from bronchial epithelial [10] and inflammatory cell [16] and may contribute in airway remodeling as a paracrine and autocrine mediator. Previous studies reported that normal lung fibroblasts express muscarinic receptors and directly respond to cholinergic stimulation, including proliferation and collagen synthesis through ERK1/2 activation [12–14]. The cholinergic agonist, relying on Rho kinase and autocrine TGF-β1 release, also induced fibroblast-to-myofibroblast transition and α-SMA expression [15]. An in vivo study revealed that anticholinergics inhibit lipopolysaccharide-induced small airway fibrosis in guinea pigs [31].

Collagen gel contraction assay is an in vitro fibrosis model developed to assess the regulation of contractile stress fiber, generally indicated as α-SMA, production from activated myofibroblasts. When differentiated myofibroblasts are cultured in such a collagen gel, the gels are contracted by the traction force, suggesting the contribution of fibrosis and abnormal tissue architecture [32]. In this study, we demonstrated that the cholinergic agent, CCh, augmented fibroblast-mediated collagen gel contraction. Compared to lung fibroblasts from non-smokers, parenchymal lung fibroblasts obtained from patients with mild to moderate COPD showed higher sensitivity to CCh or TGF-β1 in the collagen gel contraction assay. Moreover, fibroblasts obtained from patients with COPD showed higher response to CCh stimulating TGF-β1 release than those from non-smokers. These findings suggest that acetylcholine and the subsequent TGF-β1 autocrine loop play an important role in the regulation of small airway fibrosis in patients with COPD. A similar study reported that acetylcholine-induced cell proliferation was enhanced in lung fibroblasts from patients with COPD compared to cells from healthy non-smokers and healthy smokers, and muscarinic receptors were highly expressed in fibroblasts from patients with COPD compared to that in fibroblasts from controls [33]. However, we could not demonstrate different expression levels of muscarinic receptors in mild to moderate COPD fibroblasts compared to non-smoker fibroblasts (data not shown), suggesting different mechanisms of action.

ChAT, the synthase for acetylcholine, is expressed in airway epithelial cells, airway smooth muscle cells, and fibroblasts as well as in inflammatory cells such as macrophages, lymphocytes, and granulocytes [16]. Furthermore, epithelial and inflammatory cells release acetylcholine [16], but that from the other cell types remains to be determined. Thus, we measured the level of acetylcholine in the medium of cultured fibroblasts. However, acetylcholine was not detectable in the culture supernatant of fibroblasts from patients with COPD or non-smokers as well as of HFL1 cells. GLY alone did not show any effect on fibroblasts-mediated gel contraction and the cholinesterase inhibitor also could not potentiate collagen gel contraction by fibroblasts (data not shown), indicating that endogenous acetylcholine may not be involved in this mechanism. Consistently, Profita et al. reported that acetylcholine autocrine mechanism generated by ChAT was not involved in lung fibroblast proliferation [33]. In addition, ChAT expression and acetylcholine release were identified from large airways, but not from lung parenchyma, in ovalbumin-sensitized guinea pigs [9]. These findings indicated that lung parenchymal fibroblasts do not synthesize detectable amounts of acetylcholine although they do express ChAT.

ERK5, an atypical member of the MAPK family, plays an important role in hypertrophic remodeling of cardiomyocytes by enhancing cell viability and in chronic glomerulonephritis by increasing ECM deposition [34]. BIX02189, a pharmacological inhibitor of MEK5/ERK5, improved both lung fibrosis and survival rate in the bleomycin-induced lung fibrosis model [17]. García-Hoz et al. revealed that CCh induces the formation of Gαq/protein kinase C (PKC) ζ and MEK5 protein complexes to trigger ERK5 activation in mouse embryonic fibroblasts [35]. Badshah et al. reported that TGF-β1 mediates ERK5 activation in human podocytes without the involvement of the small GTPase Ras [36]. In the present study, CCh and TGF-β1 induced ERK5 phosphorylation in lung fibroblasts, and BIX02189 attenuated CCh- or TGF-β1-induced gel contraction and α-SMA expression. Furthermore, levels of basal ERK5 expression and CCh-induced ERK5 phosphorylation were higher in fibroblasts obtained from patients with COPD than in fibroblasts obtained from non-smokers, suggesting that ERK5 signaling was involved in developing airway fibrosis in COPD.

In clinical studies, inhalation of a combination of GLY and IND showed additive effects in terms of FEV1 between 5 min and 3 h post-inhalation in patients with moderate-to-sever COPD, with a synergistic effect at 15 min post-inhalation compared to the administration of either drug alone [37]. Previous studies using isolated human airway demonstrated that aclidinium and formoterol showed synergistic inhibition of airway smooth muscle tone induced by acetylcholine from 0 to 6 h when administered at low concentrations [38]. In the current study, we report for the first time that the GLY and IND combination has an additive inhibitory effect on fibroblast-mediated gel contraction in response to CCh or TGF-β1. We set the concentrations of GLY and IND in this experiment using plasma pharmacokinetics from a clinical trial; however, the actual concentration of these compounds in the airway is unclear. To verify the desired effectiveness and safety of GLY, IND, and BIX02189 in preventing airway fibrosis for clinical use, computational simulation techniques, such as fluidic dynamics, are needed to calculate the drug concentration in the peripheral airway [39].

## Conclusion

Our results demonstrate that GLY and IND combination inhibits lung fibroblast-mediated collagen gel contraction and α-SMA expression in response to CCh or TGF-β1. CCh induced TGF-β1 self-sustaining signaling loops by potentiating ERK5 signaling, which was suppressed by GLY and IND. Notably, the magnitudes of CCh- and TGF-β1-induced fibrotic activities were greater in fibroblasts isolated from mild to moderate COPD subjects than in those from non-smokers, suggesting the substantial roles of CCh- and TGF-β1 in pathogenesis of airway fibrosis. Although the present study was performed under controlled experimental settings, ERK5 might be a novel therapeutic target for airway fibrosis and the inhaled GLY and IND combination therapy may be useful for early intervention to prevent the development of airway fibrosis in COPD.

## Abbreviations

ALK5: Activin receptor–like kinase 5
CCh: Carbachol
ChAT: Choline-acetyltransferase
COPD: Chronic obstructive pulmonary disease
ELISA: Enzyme-linked immunosorbent assay
ERK1/2: Extracellular signal-regulated kinase 1/2
ERK5: Extracellular-signal-regulated kinase 5
FEV1: Forced expiratory volume in one second
FVC: Forced vital capacity
GLY: Glycopyrronium
IND: Indacaterol
LABA: Long-acting β2 adrenergic receptor agonist
LAMA: Long-acting muscarinic receptor antagonist
LC-MS/MS: Liquid chromatography tandem-mass spectrometry
MAPK: Mitogen-activated protein kinase
TGF-β1: Transforming growth factor-β1
α-SMA: α-smooth muscle actin
